# Mucormycosis in intensive care unit: surgery is a major prognostic factor in patients with hematological malignancy

**DOI:** 10.1186/s13613-020-00673-9

**Published:** 2020-06-08

**Authors:** Johanna Claustre, Romaric Larcher, Thomas Jouve, Anne-Sophie Truche, Saad Nseir, Julien Cadiet, Yoann Zerbib, Alexandre Lautrette, Jean-Michel Constantin, Pierre-Emmanuel Charles, Cedric Daubin, Remi Coudroy, Jean Dellamonica, Laurent Argaud, Pierre Phelouzat, Damien Contou, Juliette Pocquet, Guillaume Voiriot, Jean-Christophe Navellou, Pierre Lavagne, Michel Durand, Muriel Cornet, Carole Schwebel, Nicolas Terzi

**Affiliations:** 1 Service de Pneumologie, CH Annecy Genevois, 1 Avenue de l’hôpital, 74370 Epagny Metz-Tessy, France; 2 Médecine Intensive Réanimation, CHU Montpellier, Montpellier, France; 3 Université Grenoble Alpes, Grenoble, France; 4 Service Hospitalo-universitaire de Néphrologie, CHU Grenoble Alpes, Grenoble, France; 5 Médecine Intensive Réanimation, CHU Grenoble Alpes, Grenoble, France; 6 Réanimation Médicale, CHU Lille, Lille, France; 7 Service de médecine intensive Réanimation, CHU Nantes, Nantes, France; 8 Réanimation Chirurgicale, Amiens, France; 9 Réanimation CHU Clermont-Ferrand, Clermont-Ferrand, France; 10 Médecine intensive Réanimation, CHU Dijon, Dijon, France; 11 Réanimation médicale, CHU Caen, Caen, France; 12 Réanimation médicale, CHU Poitiers, Poitiers, France; 13 Médecine Intensive Réanimation, CHU Nice, Université Côte d’Azur, Nice, France; 14 Réanimation Médicale, CHU Lyon, Lyon, France; 15 Réanimation Médicale, CHU Rennes, Rennes, France; 16 Réanimation polyvalente, CH Argenteuil, Argenteuil, France; 17 Médecine intensive Réanimation, CHU Tours, Tours, France; 18 Réanimation médico-chirurgicale, CHU Tenon, Paris, France; 19 Réanimation médicale, CHU Besançon, Besançon, France; 20 Réanimation Polyvalente Chirurgicale, CHU Grenoble Alpes, Grenoble, France; 21 Réanimation Cardio-vasculaire et Thoracique, CHU Grenoble Alpes, Grenoble, France; 22 Laboratoire de Mycologie-Parasitologie, CHU Grenoble Alpes, Grenoble, France

**Keywords:** Mucormycosis, Hematological malignancy, Intensive care unit, Surgery, Prognostic factors

## Abstract

**Background:**

Mucormycosis is an invasive fungal infection, with an increasing incidence especially in patients with hematological malignancies. Its prognosis is poor because of its high invasive power and its intrinsic low susceptibility to antifungal agents. We aimed to describe the epidemiology of mucormycosis in intensive care units (ICU) and evaluate the outcomes. We performed a retrospective multi-center study in 16 French ICUs between 2008 and 2017. We compared the patients who survived in ICU and the patients who did not to identify factors associated with ICU survival. Then, we focused on the subgroup of patients with hematological malignancies.

**Results:**

Mucormycosis was diagnosed in 74 patients during the study period. Among them, 60 patients (81%) were immunocompromised: 41 had hematological malignancies, 9 were solid organ transplant recipients, 31 received long-term steroids, 11 had diabetes, 24 had malnutrition. Only 21 patients survived to ICU stay (28.4%) with a median survival of 22 days (Q1–Q3 = 9–106) and a survival rate at day 28 and day 90, respectively, of 35.1% and 26.4%. Survivors were significantly younger (*p *= 0.001), with less frequently hematological malignancies (*p *= 0.02), and less malnutrition (*p *= 0.05). Median survival in patients with hematological malignancies (*n* = 41) was 15 days (Q1–Q3 = 5–23.5 days). In this subgroup, curative surgery was a major factor associated with survival in multivariate analysis (odds ratio = 0.71, [0.45–0.97], *p *< 0.001).

**Conclusion:**

Overall prognosis of mucormycosis in ICU remains poor, especially in patients with hematological malignancies. In this subgroup of patients, a therapeutic strategy including curative surgery was the main factor associated with survival.

## Background

Mucormycosis is an invasive fungal infection, due to filamentous fungi of the mucorales type that is ubiquitous in the environment. Diagnostic criteria of mucormycosis were defined by the European Organization for Research and Treatment of Cancer/Invasive Fungal Infections Cooperative Group and the National Institute of Allergy and Infectious Diseases Mycoses Study Group (EORTC/MSG) in 2008 and are based on the association of host criteria (facultative), clinical criteria, and mycologic or histologic criteria [1]. The main known risk factors of mucormycosis are underlying conditions impairing innate immunity: allogenic hematopoietic stem cell transplantation (HSCT), chemotherapy, diabetes, long-term steroids or immunosuppressive treatments, solid organ transplantation (SOT) [2–4]. Some conditions, like multiple trauma, extensive burns [5] or some healthcare procedures [6] are also at risk of mucormycosis.

Even if the incidence of mucormycosis is quite low, it is currently increasing [3, 5]. In allogenic HSCT recipients and SOT recipients, mucormycosis reaches nowadays 7% of all invasive fungal infections, third in rank after invasive candidiasis and aspergillosis [7, 8]. Numerous factors can explain this increasing incidence. First, the number of immunocompromised patients is increasing [2, 9]. In this population, many patients accumulate different risk factors like diabetes or malnutrition [2]. Moreover, the development of new therapeutic strategies, especially the larger use of antifungal drugs that are ineffective against mucorales, such as voriconazole, can also constitute a new risk factor of mucormycosis [10, 11].

Because of the high angio-invasive power of mucorales facilitating fungal dissemination and tissular necrosis, and their low susceptibility to antifungal agents, the short-term vital and functional prognosis of mucormycosis is poor. According to the profile of the patients, mortality ranges between 16% and 73%. The highest mortality is observed in immunocompromised population, namely patients from onco-hematology [5, 9, 10].

For a decade, various studies aimed to describe the epidemiology and prognosis of mucormycosis, but most studies, published before 2010, were descriptive, retrospective and single-center studies, or only case series [2, 5, 9, 12–14]. None of these studies focused on the most severe mucormycosis, namely patients admitted in intensive care units (ICU).

We aimed to describe the epidemiology of mucormycosis in French ICUs and to evaluate the outcomes, to highlight the most important determinants of the therapeutic strategy, especially in patients with hematological malignancies.

## Methods

We performed a multi-center and retrospective analysis in 16 different French medical centers (tertiary university hospitals or general hospitals) between January 2008 and December 2017.

### Data collection

During the study period, 74 patients were diagnosed as having probable or proven mucormycosis. Cases were identified through a combination of pathology laboratory information systems and hospital coding records. Patient demographic characteristics, comorbidities and predisposing factors of mucormycosis were recorded as well as site(s) of infection, microbiological results, antifungal therapies, other therapeutic management during ICU stay, and evolutive clinical data.

We compared 2 groups: the group of patients who survived in ICU (21 patients) and the group of patients who did not (53 patients). Because of the heterogeneity of the patients and in order to limit potential confusing bias, we secondarily focused on the patients with hematological malignancies (41 patients) as this underlying condition appeared to be a major risk factor of mortality.

### Definitions

Only proven or probable infections were included. Proven or probable mucormycosis were defined according to clinical, biological, radiological and histological findings, and following the EORTC/MSG definitions for invasive fungal diseases [1]. The data were reviewed by two independent physicians.

Disseminated infection was defined as mucormycosis involving 2 or more non-contiguous sites [2].

Malnutrition was defined according to the 2015 European Society of Clinical Nutrition and Metabolism consensus statement as follows: body mass index (BMI, kg/m(2)) < 18.5, or the combined finding of unintentional weight loss (mandatory) and a reduced BMI. Weight loss could be either > 10% of habitual weight indefinite of time, or > 5% over 3 months. Reduced BMI is < 20 or < 22 kg/m^2^ in subjects younger and older than 70 years, respectively.

### Microbiological diagnosis

Clinical specimens were examined by experienced microbiologists and histopathologists in each participating center using local institutional protocols. Laboratory-based diagnoses included direct fungal stain, fungal culture, histopathology, and, in some cases, panfungal PCR. In few cases, fungal cultures remained negative despite positive histopathology or PCR. Identification of mucorales species was then impossible, mucorales were then designated by genus or as “unclassified mucorales” when genus identification was impossible.

### Statistical analysis

Quantitative data were expressed as mean and standard deviation (SD) or median and 1st and 3rd quartiles (Q1–Q3). Comparison of baseline data was performed by unpaired Student’s t test. Data exhibiting non-normal distribution were analyzed using nonparametric unpaired statistical tests (Mann–Whitney U test). Categorical variables were expressed as percentages and were compared with the Chi square test or the Fisher exact test when appropriate. A *p* value less than 0.05 was considered to be statistically significant.

Potential risk factors for death in the ICU were first studied using univariate analyses. Then variables mainly associated with death in the ICU (*p *< 0.05) were introduced for multivariate modeling by logistic regression. Because of the limited size of the cohort, the number of covariables was intentionally reduced to clinically relevant covariables: age, surgery, presence of allogenic HSCT and prognostic scores.

Data were analyzed using R (R Foundation for Statistical Computing, Vienna, Austria; http://www.R-project.org) statistical software.

### Ethical considerations

Patients were informed about the study through an information letter by each participating center. Their data were included in the study in absence of opposition. This study was approved by the ethics committees (Comité consultatif sur le traitement de l’information en matière de recherche dans le domaine de la santé and Commission nationale Informatique et Libertés) and was registered in Clinicaltrial.gov with the identifier NCT03387696.

## Results

### Demographic characteristics

During the study period, 74 patients with mucormycosis were included from 16 French ICU. Fifty-three (71.6%) of the 74 patients came from a medical ICU. The mean age was 51.0 (SD = 16.6). Most of the patients were immunocompromised: 41 patients (55.4%) had hematological malignancies, 39 patients (52.7%) had at least 2 factors altering their immunity, while we did not find any immunological predisposing factor in 13 patients, mainly among patients with trauma (Table 1).

**Table 1:** Demographic and infection characteristics of the 74 cases of zygomycosis

Demographic characteristics	Study population (n=74)	Survivors (n=21)	Non-survivors (n=53)	*p*-value
Age, years, mean (SD)^a^	50.6 (16.6)	39.5 (17.5)	55.0 (14.1)	*0.001*
Sex-ratio (H/F)	1.96	1.63	2.12	0.78
Underlying disease				
Hematological malignancies, n (%)	41 (55.4)	7 (33.3)	34 (64.2)	*0.02*
Acute myeloid leukemia, n (%)	15 (20.3)	4 (19.0)	11 (20.8)	1
Acute lymphoblastic leukemia, n (%)	10 (13.5)	2 (9.5)	8 (15.1)	0.71
Lymphoma, n (%)	8 (10.8)	0 (0)	8 (15.1)	0.10
Multiple myeloma, n (%)	2 (2.7)	0 (0)	2 (3.8)	1
Myelodysplastic syndrome, n (%)	2 (2.7)	0 (0)	2 (3.8)	1
Other, n (%)	4 (5.4)	1 (4.8)	3 (5.7)	0.84
Allogenic HSCT, n (%)	16 (21.6)	1 (4.8)	15 (28.3)	*0.03*
Autologous HSCT, n (%)	7 (9.5)	1 (4.8)	6 (11.3)	0.67
Solid malignancies, n (%)	4 (5.4)	2 (9.5)	2 (3.8)	0.32
SOT, n (%)	9 (12.2)	2 (9.5)	7 (13.2)	1
Inflammatory disease, n (%)	2 (2.7)	1 (4.8)	1 (1.9)	0.49
Systemic steroids, n (%)	30 (40.5)	2 (9.5)	28 (52.8)	*< 0.001*
Diabetes, n (%)	11 (14.9)	0 (0)	11 (20.8)	*0.03*
Other condition, n (%)				
Chronic alcoholism, n (%)	8 (10.8)	2 (9.5)	6 (11.3)	1
Malnutrition, n (%)	24 (32.4)	3 (14.3)	21 (39.6)	*0.05*
Drug addiction, n (%)	1 (1.4)	1 (4.8)	0 (0)	0.28
Chronic renal failure, n (%)	7 (9.5)	0 (0)	7 (13.2)	0.18
Respiratory disease, n (%)	15 (20.3)	5 (23.8)	10 (18.9)	0.75
Cirrhosis, n (%)	1 (1.4)	1 (4.8)	0 (0)	0.28
Trauma, n (%)	7 (9.5)	5 (23.8)	2 (3.8)	*0.017*
Burn, n (%)	3 (4.1)	1 (4.8)	2 (3.8)	1
Previous antifungal therapy, n (%)	30 (40.5)	5 (23.8)	25 (47.2)	0.07
Number of predisposing factors				*0.003*
0, n (%)	13 (17.6)	8 (38.1)	5 (9.4)	
1, n (%)	21 (28.4)	9 (42.9)	12 (22.6)	
≥ 2, n (%)	31 (41.9)	4 (19.0)	27 (50.9)	
Site of infection				
Lungs, n (%)	29 (39.2)	8 (38.1)	21 (39.6)	1
Skin, n (%)	15 (20.3)	7 (33.3)	8 (15.1)	0.11
Sinus and ENT infection, n (%)	1 (1.35)	1 (4.8)	0 (0)	0.28
Abdomen, n (%)	1 (1.35)	0 (0)	1 (1.9)	1
Bones, n (%)	1 (1.35)	1 (4.8)	0 (0)	0.28
Infection of contiguous sites, n (%)	13 (17.6)	4 (19.0)	9 (17.0)	1
Disseminated infection, n (%)	14 (18.9)	0 (0)	14 (26.4)	*0.007*
Isolated fungi				*0.04*
*Rhizopus* genus, n (%)	22 (29.7)	2 (9.5)	19 (35.8)	
*R. oryzae*, n (%)	5 (6.8)			
*R. arrhizus*, n (%)	2 (2.7)			
*R. microspores*, n (%)	1 (1.4)			
*Rhizopus sp.*, n (%)	14 (18.9)			
*Mucor* genus, n (%)	10 (13.5)	5 (23.8)	5 (9.4)	
*M. circinelloides*, n (%)	3 (4.1)			
*Mucor sp.*, n (%)	7 (9.6)			
*Rhizomucor* genus, n (%)	14 (18.9)	2 (9.5)	12 (22.6)	
*R. pusillus*, n (%)	3 (4.1)			
*R. miehei*, n (%)	1 (1.4)			
*Rhizomucor* sp., n (%)	10 (13.5)			
*Lichtheimia* genus, n (%)	19 (25.7)	8 (38.1)	11 (20.8)	
*L. corymbifera*, n (%)	14 (18.9)			
*Lichtheimia* sp., n (%)	5 (6.8)			
*Cunninghamella* genus, n (%)	1 (1.4)	0 (0)	1 (1.9)	
*C. bertholletiae*, n (%)	1 (1.4)			
Unclassified mucorales, n (%)	9 (12.2)	4 (19.0)	5 (9.4)	
Severity of infection				
SAPS2, mean (SD) ^a^	52 (22)	36 (18)	59 (19)	*< 0.001*
SOFA, mean (SD) ^a^	9 (4)	6 (3)	10 (4)	*< 0.001*
Respiratory	2.0 (1.2)	1.3 (1.3)	2.2 (1.1)	*0.02*
Cardiovascular	1.7 (1.8)	1.0 (1.4)	2.0 (1.8)	*0.01*
Renal	1.1 (1.2)	0.7 (1.0)	1.3 (1.3)	*0.04*
Hematological	2.0 (1.4)	1.0 (1.2)	2.4 (1.3)	*< 0.001*
Neurological	1.2 (1.5)	0.9 (1.4)	1.3 (1.5)	0.35
Liver	0.8 (0.9)	0.6 (0.9)	0.9 (0.9)	0.24

Italic values indicate significance of *p*-value (*p* < 0.05)

*ENT* ear-nose-throat, *HSCT* hematopoietic stem cell transplantation, *SAPS2* simplified Acute Physiological Score 2, *SOFA* sequential organ failure assessment, *SOT* solid organ transplantation

^a^Results are expressed as mean (SD) and compared with Student’s t test

### Characteristics of the infection

Forty-eight patients had a proven mucormycosis (64.9%) and 26 patients had a probable mucormycosis (35.1%). Mucormycosis mainly involved the lungs (39.2%), and the skin (20.3%). Probable mucormycosis were mainly pulmonary fungal infections. Infection involved several organs in 36.5% of the cases. In the 15 cases of disseminated infection, lungs were involved in 10 patients, skin in 8 patients, abdomen in 7 patients, central nervous system (CNS) in 5 patients and sinus in 1 patient. Mucormycosis was diagnosed on fungal cultures in 30 cases (40.5%), on histology in 19 cases (25.7%) and on PCR in 25 patients (33.8%). The isolated fungi were from *Rhizopus* genus in 22 cases (29.7%), *Lichtheimia* genus in 19 cases (25.7%), *Rhizomucor* genus in 14 cases (18.9%), *Mucor* genus in 9 cases (12.2%), *Cunninghamella* genus in one case (1.4%). The last 10 cases (13.5%) remained “unclassified mucorales” (Table 1). The median time of positive diagnosis of mucormycosis after the onset of the symptoms was 7 days (Q1–Q3 = 4–17.3); in 6 patients, the diagnosis was performed after the death. The median time between the beginning of the symptoms and ICU admission was 4.5 days (Q1–Q3 = 1–10.8). Although clinical symptoms were already present, mucormycosis diagnosis was unknown at the ICU admission in 60 patients (81.1%). In 12 patients (16.2%), symptoms appeared during ICU hospitalization, mainly in patients with trauma.

### Survival analysis

Among the 74 patients, only 21 patients survived to ICU stay, with an overall survival in ICU of 28.4%. Median survival in the study group was 22 days (Q1–Q3 = 9–106), with a survival rate at day 28 and day 90, respectively, of 35.1% and 26.4%.

When comparing the survivors of ICU stay with non-survivors, we observed numerous striking differences in the univariate analysis in terms of demographic characteristics (Table 1). The demographic characteristics independently associated with a higher mortality were the presence of a hematological malignancy (odds ratio (OR) = 1.31 [1.13–1.48], *p *= 0.003), malnutrition (OR = 1.24 [1.07–1.42], *p *= 0.02) and age (OR = 1.01 [1.01–1.02] per year after median age, *p *< 0.001). All patients with a disseminated infection died in ICU (*p* = 0.007). Infections due to *Rhizopus* and *Rhizomucor* genera had also a worse prognosis (*p *= 0.04). The main cause of ICU admission was acute respiratory failure in both groups. The cause of ICU admission was not significantly associated with survival (*p *= 0.11). SOFA score was significantly higher in the non-survivor group (*p *< 0.001), in which respiratory failure, hemodynamic failure, renal failure and hematological failure were more represented (*p *< 0.05; Table 1).

Regarding therapeutic strategy (Table 2), efficient medical treatment was started with a mean delay after the beginning of the symptoms of 8.2 (SD = 7.6) days in the survivor group and 10.1 (SD = 11.3) days in the non-survivor group (*p *= 0.45). No difference was observed in survival according to antifungal treatment strategy. Forty-nine patients (81.7%) received L-AmB at the dosage of 5 mg/kg and 21 received a higher dosage (18.3%) without any significant difference in the prognosis (*p *= 0.47). Twelve patients (16.2%) received a combination of antifungal treatments: 8 L-AmB with caspofungin and 4 L-AmB with posaconazole without difference in prognosis between patients treated with L-AmB alone or in combination (*p *= 0.52). Strategies including a surgical therapeutic management was associated with a better survival in univariate analysis (*p *= 0.03). Characteristics of curative surgery are detailed in Table 2. Only lung resection was associated with survival (*p* = 0.002). Characteristics of patients who underwent surgery are compared with those who did not in Table 3. Briefly, a therapeutic strategy including surgery was performed in patients with less diabetes (*p *= 0.05), less thrombopenia (*p *= 0.02), with a lower Simplified Acute Physiological Score 2 (SAPS 2) score (*p *= 0.01) and a lower Sequential Organ Failure Assessment (SOFA) score (*p *= 0.001). The median delay between the beginning of the symptoms and therapeutic surgery was 12 days (Q1–Q3 = 6–20.5) without any significant difference between survivors and non-survivors (*p *= 0.10).

**Table 2:** Characteristics of ICU lengh stay and therapeutic strategy

	Number of patients (%)	Survivors (*n* = 21)	Non-survivors (*n* = 53)	P-value
Type of ICU				0.11
Medical, *n* (%)	53 (71.6)	13 (61.9)	40 (75.5)	
Surgical, *n* (%)	13 (17.6)	3 (14.3)	10 (18.9)	
Polyvalent, *n* (%)	8 (10.8)	5 (23.8)	3 (5.7)	
Year of admission				0.70
Antifungal treatment				
Treatment including L-AmB	56 (75.7)	19 (90.5)	37 (69.8)	0.08
L-AmB alone, *n* (%)	44 (59.5)	14 (66.7)	30 (56.6)	0.60
Association of L-AmB + other drug, *n* (%)	12 (16.2)	5 (23.8)	7 (13.2)	0.30
Triazole drug alone, *n* (%)	4 (5.4)	1 (4.8)	3 (5.7)	1
Echinocandine alone, *n* (%)	1 (1.4)	0 (0)	1 (1.9)	1
None, *n* (%)	13 (17.6)	1 (4.8)	12 (22.6)	0.09
Delay of efficient antifungal treatment, mean (SD)^a^	9.5 (9.9)	8.2 (7.6)	10.1 (11.3)	0.45
Interventions				
Surgical intervention, n (%)	27 (36.5)	12 (57.1)	15 (28.3)	0.03
Cutaneous resection	11 (14.9)	3 (14.3)	8 (15.1)	1
Lung resection	7 (9.5)	6 (28.6)	1 (1.9)	*0.002*
Amputation	2 (2.7)	2 (9.5)	0	0.08
ENT resection	4 (5.4)	1 (4.8)	3 (5.7)	1
Abdominal resection	3 (4.1)	0	3 (5.7)	0.55
Vasopressors, *n* (%)	53 (71.6)	9 (42.9)	44 (83.0)	0.001
Mechanical ventilation, *n* (%)	66 (89.2)	14 (66.7)	52 (98.1)	< 0.001
Renal replacement therapy, *n* (%)	31 (41.9)	5 (23.8)	26 (49.1)	0.07

Italic values indicate significance of *P*-value (*P* < 0.05)

*L-AmB* liposomal amphotericin B

^a^Results are expressed as mean (SD) and compared with Student’s t test

**Table 3:** Comparison of the 74 mucormycosis according to the surgical status

	Study population (n = 74)	Surgery (n = 27)	No surgery (n = 47)	*P*-*value*
Age, years, mean (SD)^a^	50.6 (16.6)	49.2 (19.6)	51.5 (14.8)	0.60
Sex-ratio (H/F)	1.96	1.1	2.92	0.08
Underlying disease				
Hematological malignancies, *n* (%)	41 (55.4)	12 (44.4)	29 (61.7)	0.23
Acute myeloid leukemia, *n* (%)	15 (20.3)	5 (18.5)	10 (21.3)	1
Acute lymphoblastic leukemia, *n* (%)	10 (13.5)	3 (11.1)	7 (14.9)	0.74
Lymphoma, *n* (%)	8 (10.8)	3 (11.1)	5 (10.6)	1
Multiple myeloma, *n* (%)	2 (2.7)	0	2 (4.3)	0.53
Myelodysplastic syndrome, *n* (%)	2 (2.7)	0	2 (4.3)	0.53
*Other, n* (%)	4 (5.4)	1 (3.7)	3 (6.4)	1
Allogenic HSCT, *n* (%)	16 (21.6)	3 (11.1)	13 (27.7)	0.14
Autologous HSCT, *n* (%)	7 (9.5)	1 (3.7)	6 (12.8)	0.41
Solid malignancies, *n* (%)	4 (5.4)	1 (3.7)	3 (6.4)	1
SOT, *n* (%)	9 (12.2)	4 (14.8)	5 (10.6)	0.72
Inflammatory disease, *n* (%)	2 (2.7)	1 (3.7)	1 (2.1)	1
Systemic steroids, *n* (%)	30 (40.5)	8 (29.6)	22 (46.8)	0.15
Diabetes, *n* (%)	11 (14.9)	1 (3.7)	10 (21.3)	*0.05*
Other condition, n (%)				
*Chronic alcoholism,* n (%)	8 (10.8)	3 (11.1)	5 (10.6)	1
*Malnutrition, n* (%)	24 (32.4)	10 (37.0)	14 (29.8)	0.70
*Drug addiction, n* (%)	1 (1.4)	0	1 (2.1)	1
*Chronic renal failure, n* (%)	7 (9.5)	3 (11.1)	4 (8.5)	1
*Respiratory disease, n* (%)	15 (20.3)	2 (7.4)	13 (27.7)	0.13
*Cirrhosis, n* (%)	1 (1.4)	0	1 (2.1)	1
*Trauma, n* (%)	7 (9.5)	2 (7.4)	5 (10.6)	1
*Burn, n* (%)	3 (4.1)	2 (7.4)	1 (2.1)	0.55
Previous antifungal therapy, *n* (%)	30 (40.5)	7 (25.9)	23 (48.9)	0.09
Site of infection				
Lungs, *n* (%)	29 (39.2)	5 (18.5)	24 (51.1)	0.01
Skin, *n* (%)	15 (20.3)	8 (29.6)	7 (14.9)	0.22
Sinus and ENT infection, *n* (%)	1 (1.35)	1 (1.4)	0	0.36
Abdomen, *n* (%)	1 (1.35)	1 (1.4)	0	0.36
Bones, n (%)	1 (1.35)	0	1 (2.1)	1
Infection of contiguous sites, *n* (%)	13 (17.6)	7 (25.9)	6 (12.8)	0.26
Disseminated infection, *n* (%)	14 (18.9)	4 (14.8)	10 (21.3)	0.55
Isolated fungi				*0.02*
*Rhizopus* genus, *n* (%)	22 (29.7)	7 (25.9)	15 (31.9)	
*Mucor* genus, *n* (%)	10 (13.5)	2 (7.4)	8 (17.0)	
*Rhizomucor* genus, *n* (%)	14 (18.9)	4 (14.8)	10 (21.3)	
*Lichtheimia* genus, *n* (%)	19 (25.7)	7 (25.9)	12 (25.5)	
*Cunninghamella* genus, n (%)	1 (1.4)	0	1 (2.1)	
Unclassified mucorales, *n* (%)	9 (12.2)	8 (29.6)	1 (2.1)	
Year of admission				0.53
Severity of infection				
SAPS2, mean (SD)^a^	52 (22)	44.6 (17.5)	56.6 (22.5)	*0.01*
SOFA, mean (SD)^a^	9 (4)	7.0 (3.3)	9.9 (3.7)	*0.001*
Respiratory	2.0 (1.2)	1.5 (1.3)	2.2 (1.1)	*0.01*
Cardiovascular	1.7 (1.8)	1.4 (1.6)	1.9 (1.8)	0.18
Renal	1.1 (1.2)	1.2 (1.2)	1.1 (1.2)	0.63
Hematological	2.0 (1.4)	1.4 (1.2)	2.3 (1.5)	*0.006*
Neurological	1.2 (1.5)	0.8 (1.3)	1.4 (1.6)	0.08
Liver	0.8 (0.9)	0.6 (0.8)	0.9 (0.9)	0.17
Biological abnormalities				
TP < 50%, *n* (%)	13 (17.6)	2 (7.41)	11 (23.4)	0.12
Thrombocytes < 50G/L, *n* (%)	27 (36.5)	5 (18.5)	22 (46.8)	*0.02*
Leucocytes < 1G/L, *n* (%)	23 (31.1)	6 (22.2)	17 (36.2)	0.32
Interventions				
Vasopressors	53 (71.6)	18 (66.7)	35 (74.5)	0.65
Mechanical ventilation	66 (89.2)	21 (77.8)	45 (95.7)	*0.05*
Renal replacement therapy	31 (41.9)	14 (51.9)	17 (36.2)	0.23
Delay of efficient antifungal treatment, mean (SD)^a^	9.5 (9.9)	10.2 (8.7)	8.9 (11.2)	0.64

Italic values indicate significance of *P*-value (*P* < 0.05)

*ENT* ear-nose-throat, *HSCT* hematopoietic stem cell transplantation; *SAPS2* simplified Acute Physiological Score 2, *SOFA* sequential organ failure assessment, *SOT* solid organ transplantation

^a^Results are expressed as mean (SD) and compared with Student’s t test

### Patients with hematological malignancies

As the presence of a hematological malignancy was independently associated with a worse prognosis, we focused on this group of patients. Hematological malignancies are described in Table 1. In the 16 patients who received an allogenic HSCT, 11 patients had a Grade ≥ 2 chronic graft-versus-host disease (Additional file 1: Table S1).

Patients with hematological malignancies were more likely to have mucormycosis involving the lungs, the abdomen and/or the CNS and less likely to have sinus infection compared to the other patients (*p *= 0.05). The isolated fungus was more frequently from *Rhizopus* genus or *Rhizomucor* genus and less frequently from *Lichtheimia* genus (*p *= 0.03) (Additional file 1: Table S2). Fourteen patients (34.1%) were exposed to fluconazole or voriconazole in the 3 months before the first symptoms of mucormycosis.

**Fig. 1:**
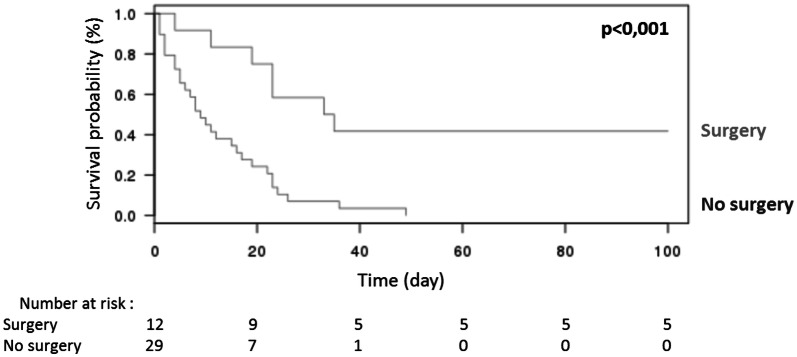
Zygomycosis survival in the subgroup of patients with hematological malignancies: comparison between those who benefited from surgery (Surgery) and those who did not (no Surgery), with number of patients at risk

In the 41 patients with hematological malignancies, median survival was worse than in the other patients, evaluated at 15 days (Q1–Q3 = 5–23.5), with a survival rate at day 28 and day 90, respectively, of 22.0% and 14.6% (*p *= 0.001). Only 7 patients (17.1%) survived to ICU stay (Additional file 1: Table S3). The demographic characteristics associated with a poorer prognosis were age (*p *= 0.04), a previous exposure to antifungal drug in the last 3 months (*p *< 0.001) and a leukocytes level inferior to 1 G/L at ICU admission (*p *= 0.04). A therapeutic strategy including curative surgery was strongly associated with survival (*p *= 0.001) as well as prognostic scores and markers of organ failure: SAPS2 (*p *< 0.001), SOFA score (*p *= 0.002), and more precisely respiratory failure (*p *= 0.005), hemodynamic failure (*p *< 0.001), neurological failure (*p *= 0.001), need for vasopressors (*p *< 0.001), need for mechanical ventilation (*p *< 0.001) (Table 4). Characteristics of the curative surgery are detailed in Table 4: lung resection was strongly associated with survival (p < 0.001). The delay between the diagnosis of hematological malignancy and mucormycosis was not different between survivors and non-survivors (*p *= 0.66). In the multivariate analysis, therapeutic surgery was strongly associated with a better prognosis (OR = 0.71 [0.45–0.97], *p *= 0.01), after adjusting on demographic characteristics. Using the Kaplan–Meier estimator, overall survival was also better in the subgroup of patients who benefited from surgery (Fig. 1, *p* < 0.001). No difference was observed in terms of prognosis regarding the dosage of L-AmB in both groups (*p *= 1). Only 7 patients received granulocyte-colony stimulating factor, with no difference on ICU survival (*p *= 1).

**Table 4:** Characteristics of the 41 zygomycosis in patients with hematological malignancy

	Patients with hematological malignancies (*n* = 41)	Patients who survived (*n* = 7)	Patients who did not survived (*n* = 34)	*P*-*value*
Age, mean (SD)^a^	48.4 (15.0)	34.7 (16.3)	51.3 (13.3)	*0.04*
Sex-ratio	2.4	1.3	2.8	0.40
Malnutrition, *n* (%)	14 (34.1)	1 (14.3)	13 (38.2)	0.39
Diabetes, *n* (%)	7 (17.1)	0 (0)	7 (20.6)	0.32
Graft-versus-host disease, *n* (%)	12 (29.3)	1 (14.3)	11 (32.4)	0.65
Previous antifungal therapy, *n* (%)	24 (58.5)	0 (0)	24 (70.6)	*< 0.001*
Site of infection				
Lungs, *n* (%)	19 (48.8)	4 (57.1)	16 (47.1)	0.70
Skin, *n* (%)	3 (7.3)	1 (14.3)	2 (5.9)	0.44
Sinus and ENT infection, *n* (%)	1 (2.4)	1 (14.3)	0 (0)	0.17
Abdomen, *n* (%)	1 (2.4)	0 (0)	1 (2.9)	1
Bones, *n* (%)	0 (0)	0 (0)	0 (0)	1
Contiguous infection of > 1 organ, *n* (%)	6 (14.6)	1 (14.3)	5 (14.7)	1
Disseminated infection, *n* (%)	10 (24.4)	0 (0)	10 (29.4)	0.16
Isolated fungi				0.19
*Rhizopus sp.*, *n* (%)	15 (36.6)	1 (14.3)	14 (41.2)	
*Mucor sp.*, *n* (%)	2 (4.9)	0 (0)	2 (5.9)	
*Rhizomucor sp.*, *n* (%)	11 (26.8)	2 (28.6)	9 (26.5)	
*Lichtheimia sp.*, *n* (%)	7 (17.1)	1 (14.3)	6 (17.6)	
*Cunninghamella sp.*, *n* (%)	1 (2.4)	0 (0)	1 (2.9)	
Not identified zygomycetes, *n* (%)	5 (12.2)	3 (42.9)	2 (5.9)	
Severity of infection				
SAPS2, mean (SD)^a^	55.3 (21.0)	31.4 (22.4)	60.3 (21.0)	*< 0.001*
SOFA, mean (SD)^a^	9.2 (4.2)	4.0 (4.5)	10.1 (4.3)	*0.002*
Respiratory	2.0 (1.1)	0.9 (0.9)	2.3 (1.0)	*0.005*
Cardiovascular	1.3 (1.8)	0.2 (0.4)	1.5 (1.8)	*< 0.001*
Renal	0.9 (1.1)	0.7 (1.0)	0.9 (1.1)	0.61
Hematological	2.8 (1.2)	1.5 (1.6)	3.1 (1.0)	0.07
Neurological	1.0 (1.4)	0.1 (0.4)	1.2 (1.5)	*0.001*
Liver	1.0 (0.9)	0.7 (0.8)	1.1 (1.0)	0.29
Biological abnormalities				
TP < 50%, *n* (%)	7 (17.1)	0 (0)	7 (20.6)	0.32
Thrombocytes < 50G/L, *n* (%)	24 (58.5)	2 (28.6)	22 (64.7)	0.11
Leucocytes < 1G/L, *n* (%)	21 (51.2%)	1 (14.3)	20 (58.8)	*0.04*
Interventions				
Surgical intervention, *n* (%)	12 (29.3)	6 (85.7)	6 (17.6)	*0.001*
Cutaneous resection	2 (4.9)	0	2 (5.9)	1
Lung resection	5 (12.2)	5 (71.4)	0	*< 0.001*
ENT resection	3 (7.3)	1 (14.3)	2 (5.9)	0.44
Abdominal resection	2 (4.9)	0	2 (5.9)	1
Vasopressors, n (%)	27 (65.9)	0 (0)	27 (79.4)	*< 0.001*
Mechanical ventilation, n (%)	35 (85.4)	1 (14.3)	34 (100)	*<0.001*
Renal replacement, n (%)therapy	12 (29.3)	0 (0)	12 (35.3)	0.08

Italic values indicate significance of *P*-value (*P* < 0.05)

*ENT* ear-nose-throat, *SAPS2* simplified Acute Physiological Score 2, *SOFA* sequential organ failure assessment

^a^Results are expressed as mean (SD) and compared with Student’s t test

## Discussion

This is the first multi-center study aimed to describe the epidemiology and the outcome of patients admitted in ICU and presenting mucormycosis. Despite the latest advances in ICU standard of care, the global ICU mortality for mucormycosis remained very high, reaching here 71.6%, close to studies focusing on immunocompromised patients [3, 7]. Some factors appeared to be determinant in ICU survival.

### Patient and infection characteristics

We observed more fatal mucormycosis in older patients, in patients with malnutrition or with hematological malignancies. In older studies, the major prognostic factors were hematological malignancy, allogenic HSCT, diabetes or HIV infection [2, 4, 9, 17, 18]. Age was already described as a prognostic factor in one study [13]. Malnutrition, known as a risk factor of mucormycosis [19], has not been already clearly identified as a prognostic factor. As we showed, the presence of two or more predisposing factors has also clearly a negative impact on prognosis [2].

Some characteristics of the infection were of importance. First, we observed less proven mucormycosis (65%) than in other studies [26], in which the percentage of proven zygomycosis is close to 85%. In our study, the majority of the patients had pulmonary mucormycosis, with a lower rate of patients who underwent surgery (36.4%), versus 71% of the patients in Lanternier’s study [26]. Pulmonary mucormycosis, which did not undergo surgery, were classified as probable mucormycosis as the diagnosis was mainly performed on broncho-alveolar lavage [1]. However, our rate of proven mucormycosis is close to other studies [2–15].

Some infections sites have been suggested as at higher risk of mortality, like abdomen [18] or renal graft [4], but our study could not confirm these results. Interestingly, the majority of mucormycosis observed in ICU were pulmonary infections, and this site has been shown to be associated with hematological malignancies [2, 13]. Because of this confusing bias, we cannot conclude that any isolated site of infection was associated with an increased mortality. However, disseminated infections were clearly associated with a poorer prognosis. The worse prognosis of disseminated infections is well known in SOT recipients and in allogenic HSCT recipients [4, 8, 18, 20, 21]. In this study, some genera were also associated with a poorer prognosis in ICU, especially *Rhizopus* and *Rhizomucor*. This result is not concordant with previous microbiological studies showing a higher virulence in experimental model and a worse prognosis for *Cunninghamella* genus [9, 16, 18]. In our study, fungi from *Rhizopus* or *Rhizomucor* genera were more frequently found in patients with hematological malignancies. When focusing on these patients there was no significant difference in prognosis regarding the genus of mucormycosis. In these patients, rather than the causative genus, a previous exposure to antifungal therapy, was associated with a worse prognosis. Indeed, the use of antifungal prophylaxis, especially those based on voriconazole use, is a risk factor of mucormycosis [10, 19, 22]. This drug has no anti-mucorales effect and participates in selection of this pathogen. Furthermore, some preliminary studies think that exposure to voriconazole could selectively enhance mucorales virulence [23, 24].

### Treatment strategy

The two pillars of the optimal management of mucormycosis are: (i) the early introduction of amphotericin B-based therapy, as a delayed medical treatment more than 5 days after the onset of the symptoms is a strong prognostic factor of mortality [17]; and (ii) the early performance of invasive therapeutic strategies [25]. In our study, most of the patients needed critical care before the positive diagnosis of mucormycosis. In these circumstances, an undelayed empirical treatment active against mucorales, especially amphotericin B-based therapy, is of major importance [17, 22]. Concerning the management of antifungal therapy, we observed heterogeneous practices. This heterogeneity reflects that some questions are still present regarding mucormycosis standard of care. First, dosage of L-AmB was variable, in the range of 5 to 10 mg/kg/day. In patients with hematological malignancies, the current recommended dosage is 5 mg/kg/day [16]. One prospective trial recently assessed the efficacy and safety of first-line therapy with high-dose L-AmB given at 10 mg/kg/day, but this pilot study was uncontrolled and showed 40% of acute kidney injury [26]. There is currently no comparative trial aimed to determine the optimal dosage of L-AmB in this indication. Secondly, we observed the use of combination of antifungal treatments. No significant difference in prognosis was shown concerning the use of L-AmB alone or in combination with any other antifungal drug in our study, which included a low number of patients. The current level of recommendations concerning the association of different antifungal drug is low, with a Grade C [16]. Some studies suggested a benefit in survival with an association of L-AmB with posaconazole [27, 28] or echinocandin [29], but in the subgroup of patients with hematological malignancies, a recent retrospective study did not show any difference in 6-week mortality with the use of combination treatments [15]. Thirdly, the effect of isavuconazole was not evaluated in this study as only two patients received this extended-spectrum triazole. However, this new drug seems to be of very high interest in mucormycosis, as it showed a similar efficacy to amphotericin B in a case–control analysis [30].

Moreover, in our study, survival strikingly increased in patients with hematological malignancies who benefited from a curative surgical management. Even if no previous studies established the benefit of surgery specifically in patients with hematological malignancies, this finding is confirmed in numerous previous studies for more than three decades, especially for rhino-orbital or pulmonary localizations [31–33], and in other at-risk groups like diabetic patients or SOT recipients [4, 20, 32, 34]. In patients with hematological malignancies, current recommendations strongly confirm the place of an early surgical management, when feasible [16, 35]. However, frailest patients or those with major coagulopathy are not eligible for surgery, as well as those with disseminated mucormycosis. These characteristics constitute confusing biases in all studies and participate to overestimate the impact of surgery but, because of the high angio-invasive power of mucorales, surgery has to be the cornerstone of the therapeutic strategy. The optimal delay of surgery is unknown, but it should not be delayed. This study is not designed to identify which patients could or could not be operated for mucormycosis. Indeed, the feasibility of the surgery and the variables that could interfere with the decision were not evaluated. But, this study showed that, when a curative surgical management was not performed, no medium- or long-term survivors were observed in patients with hematological malignancies despite efficient medical treatment and ICU standard of care. In this subgroup of patients, when the treatment cannot be optimal, early therapeutic limitation should be discussed, to avoid extended futile invasive care.

Due to the observational and retrospective design of the study, further biases have to be acknowledged. First, the study period covered 10 years, from the publication of the last EORTC/MSG definitions for invasive fungal diseases [1]. Contrary to previous studies with a longer enrollment period [14, 15], this study period was chosen to be representative of the current diagnostic and therapeutic practices, reflecting not only the specific management of mucormycosis [16], but also the current standard of care in ICU. Secondly, patients included were very heterogeneous, especially in terms of underlying conditions, and the risk of confusing biases is high. Our goal was first to describe the wide spectrum of mucormycosis in ICU. We secondarily focused on patients with hematological malignancies in order to describe a more homogeneous subgroup, which is the one with the worst prognosis and the one who needs the more a better and earlier evaluation in order to guide the optimal treatment strategy. Thirdly, the small size of the cohort, above all of the subgroup of patients with hematological malignancy, allowed including a very limited number of covariables in the multivariate model. The covariables included in the multivariate analysis were intentionally reduced to some demographic characteristics previously identified to be associated with prognosis in the literature. Despite these numerous limitations, this study illustrates the poor short-term prognosis of patients presenting mucormycosis in ICU, and highlights some factors which could help to optimize the medical decisions.

## Conclusion

In this French multi-center cohort, the prognosis of mucormycosis in ICU remained poor, especially in older patients, in those with hematological malignancies, malnutrition, or in those who accumulate numerous predisposing factors. In patients with hematological malignancies, a treatment strategy including surgery was critical for good outcomes. In these patients, when surgery was not possible, especially in the cases of disseminated infections, the short- and medium-term prognosis was catastrophic.

## Supplementary information

**Additional file 1: Table S1.** Demographic characteristics of patients with hematological disease. **Table S2.** Characteristics of the infection of patients with hematological disease at ICU admission. **Table S3.** Characteristics of ICU management of patients with hematological disease.

## Data Availability

The datasets used and/or analyzed during the current study are available from the corresponding author on reasonable request.

## Abbreviations

CNS: Central nervous system
ENT: Ear, nose, throat
EORTC/MSG: European Organization for Research and Treatment of Cancer/Invasive Fungal Infections Cooperative Group and the National Institute of Allergy and Infectious Diseases Mycoses Study Group
HSCT: Hematopoietic stem cell transplantation
ICU: Intensive care unit
L-AmB: Liposomal amphotericin B
OR: Odds ratio
Q: Quartile
SAPS2: Simplified Acute Physiological Score 2
SD: Standard deviation
SOFA: Sequential organ failure assessment
SOT: Solid organ transplant

## References

[1] De Pauw B, Walsh TJ, Donnelly JP, Stevens DA, Edwards JE, Calandra T, Pappas PG, Maertens J, Lortholary O, Kauffman CA, Denning DW, Patterson TF, Maschmeyer G, Bille J, Dismukes WE, Herbrecht R, Hope WW, Kibbler CC, Kullberg BJ, Marr KA, Munoz P, Odds FC, Perfect JR, Restrepo A, Ruhnke M, Segal BH, Sobel JD, Sorrell TC, Viscoli C, Wingard JR, Zaoutis T, Bennett JE, European Organization for R, Treatment of Cancer/Invasive Fungal Infections Cooperative G, National Institute of A, Infectious Diseases Mycoses Study Group Consensus G (2008). ) Revised definitions of invasive fungal disease from the European Organization for Research and Treatment of Cancer/Invasive Fungal Infections Cooperative Group and the National Institute of Allergy and Infectious Diseases Mycoses Study Group (EORTC/MSG) Consensus Group. Clin Infect Dis.

[2] Lanternier F, Dannaoui E, Morizot G, Elie C, Garcia-Hermoso D, Huerre M, Bitar D, Dromer F, Lortholary O, French Mycosis Study G (2012). A global analysis of mucormycosis in France: the RetroZygo Study (2005-2007). Clin Infect Dis.

[3] Park BJ, Pappas PG, Wannemuehler KA, Alexander BD, Anaissie EJ, Andes DR, Baddley JW, Brown JM, Brumble LM, Freifeld AG, Hadley S, Herwaldt L, Ito JI, Kauffman CA, Lyon GM, Marr KA, Morrison VA, Papanicolaou G, Patterson TF, Perl TM, Schuster MG, Walker R, Wingard JR, Walsh TJ, Kontoyiannis DP (2011). Invasive non-Aspergillus mold infections in transplant recipients, United States, 2001-2006. Emerg Infect Dis.

[4] Song Y, Qiao J, Giovanni G, Liu G, Yang H, Wu J, Chen J (2017). Mucormycosis in renal transplant recipients: review of 174 reported cases. BMC Infect Dis.

[5] Bitar D, Che D, (2013) [Epidemiology of mucormycosis in metropolitan France, 1997-2010]. Medecine sciences: M/S 29 Spec No 1: 7-12. 10.1051/medsci/201329s103 23510519

[6] Rammaert B, Lanternier F, Zahar JR, Dannaoui E, Bougnoux ME, Lecuit M, Lortholary O (2012). Healthcare-associated mucormycosis. Clin Infect Dis.

[7] Neofytos D, Horn D, Anaissie E, Steinbach W, Olyaei A, Fishman J, Pfaller M, Chang C, Webster K, Marr K (2009). Epidemiology and outcome of invasive fungal infection in adult hematopoietic stem cell transplant recipients: analysis of multicenter prospective antifungal therapy (PATH) alliance registry. Clin Infect Dis.

[8] Lanternier F, Sun HY, Ribaud P, Singh N, Kontoyiannis DP, Lortholary O (2012). Mucormycosis in organ and stem cell transplant recipients. Clin Infect Dis.

[9] Roden MM, Zaoutis TE, Buchanan WL, Knudsen TA, Sarkisova TA, Schaufele RL, Sein M, Sein T, Chiou CC, Chu JH, Kontoyiannis DP, Walsh TJ (2005). Epidemiology and outcome of zygomycosis: a review of 929 reported cases. Clin Infect Dis.

[10] Trifilio SM, Bennett CL, Yarnold PR, McKoy JM, Parada J, Mehta J, Chamilos G, Palella F, Kennedy L, Mullane K, Tallman MS, Evens A, Scheetz MH, Blum W, Kontoyiannis DP (2007). Breakthrough zygomycosis after voriconazole administration among patients with hematologic malignancies who receive hematopoietic stem-cell transplants or intensive chemotherapy. Bone Marrow Trans.

[11] Trifilio S, Singhal S, Williams S, Frankfurt O, Gordon L, Evens A, Winter J, Tallman M, Pi J, Mehta J (2007). Breakthrough fungal infections after allogeneic hematopoietic stem cell transplantation in patients on prophylactic voriconazole. Bone Marrow Trans.

[12] Ruping MJ, Heinz WJ, Kindo AJ, Rickerts V, Lass-Florl C, Beisel C, Herbrecht R, Roth Y, Silling G, Ullmann AJ, Borchert K, Egerer G, Maertens J, Maschmeyer G, Simon A, Wattad M, Fischer G, Vehreschild JJ, Cornely OA (2010). Forty-one recent cases of invasive zygomycosis from a global clinical registry. J Antimicrob Chemother.

[13] Skiada A, Pagano L, Groll A, Zimmerli S, Dupont B, Lagrou K, Lass-Florl C, Bouza E, Klimko N, Gaustad P, Richardson M, Hamal P, Akova M, Meis JF, Rodriguez-Tudela JL, Roilides E, Mitrousia-Ziouva A, Petrikkos G, European Confederation of Medical Mycology Working Group on Z (2011). Zygomycosis in Europe: analysis of 230 cases accrued by the registry of the European Confederation of Medical Mycology (ECMM) Working Group on Zygomycosis between 2005 and 2007. Clin Microbiol Infect.

[14] Kennedy KJ, Daveson K, Slavin MA, van Hal SJ, Sorrell TC, Lee A, Marriott DJ, Chapman B, Halliday CL, Hajkowicz K, Athan E, Bak N, Cheong E, Heath CH, Morrissey CO, Kidd S, Beresford R, Blyth C, Korman TM, Robinson JO, Meyer W, Chen SC, Australia, New Zealand Mycoses Interest Group of the Australasian Society for Infectious D (2016). Mucormycosis in Australia: contemporary epidemiology and outcomes. Clin Microbiol Infect.

[15] Kyvernitakis A, Torres HA, Jiang Y, Chamilos G, Lewis RE, Kontoyiannis DP (2016). Initial use of combination treatment does not impact survival of 106 patients with haematologic malignancies and mucormycosis: a propensity score analysis. Clin Microbiol Infect.

[16] Tissot F, Agrawal S, Pagano L, Petrikkos G, Groll AH, Skiada A, Lass-Florl C, Calandra T, Viscoli C, Herbrecht R (2017). ECIL-6 guidelines for the treatment of invasive candidiasis, aspergillosis and mucormycosis in leukemia and hematopoietic stem cell transplant patients. Haematologica.

[17] Chamilos G, Lewis RE, Kontoyiannis DP (2008). Delaying amphotericin B-based frontline therapy significantly increases mortality among patients with hematologic malignancy who have zygomycosis. Clin Microbiol Infect.

[18] Sun HY, Singh N (2011). Mucormycosis: its contemporary face and management strategies. Lancet Infect Dis.

[19] Kontoyiannis DP, Lionakis MS, Lewis RE, Chamilos G, Healy M, Perego C, Safdar A, Kantarjian H, Champlin R, Walsh TJ, Raad II (2005). Zygomycosis in a tertiary-care cancer center in the era of Aspergillus-active antifungal therapy: a case-control observational study of 27 recent cases. J Infect Dis.

[20] Singh N, Aguado JM, Bonatti H, Forrest G, Gupta KL, Safdar N, John GT, Pursell KJ, Munoz P, Patel R, Fortun J, Martin-Davila P, Philippe B, Philit F, Tabah A, Terzi N, Chatelet V, Kusne S, Clark N, Blumberg E, Julia MB, Humar A, Houston S, Lass-Florl C, Johnson L, Dubberke ER, Barron MA, Lortholary O (2009). Zygomycosis in solid organ transplant recipients: a prospective, matched case-control study to assess risks for disease and outcome. J Infect Dis.

[21] Almyroudis NG, Sutton DA, Linden P, Rinaldi MG, Fung J, Kusne S (2006). Zygomycosis in solid organ transplant recipients in a tertiary transplant center and review of the literature. Am J Transplant.

[22] Kontoyiannis DP, Lewis RE (2011). How I treat mucormycosis. Blood.

[23] Lamaris GA, Ben-Ami R, Lewis RE, Chamilos G, Samonis G, Kontoyiannis DP (2009). Increased virulence of Zygomycetes organisms following exposure to voriconazole: a study involving fly and murine models of zygomycosis. J Infect Dis.

[24] Lewis RE, Liao G, Wang W, Prince RA, Kontoyiannis DP (2011). Voriconazole pre-exposure selects for breakthrough mucormycosis in a mixed model of Aspergillus fumigatus-Rhizopus oryzae pulmonary infection. Virulence.

[25] Jeong SJ, Lee JU, Song YG, Lee KH, Lee MJ (2015). Delaying diagnostic procedure significantly increases mortality in patients with invasive mucormycosis. Mycoses.

[26] Lanternier F, Poiree S, Elie C, Garcia-Hermoso D, Bakouboula P, Sitbon K, Herbrecht R, Wolff M, Ribaud P, Lortholary O, French Mycosis Study G (2015). Prospective pilot study of high-dose (10 mg/kg/day) liposomal amphotericin B (L-AMB) for the initial treatment of mucormycosis. J Antimicrob Chemother.

[27] van Burik JA, Hare RS, Solomon HF, Corrado ML, Kontoyiannis DP (2006). Posaconazole is effective as salvage therapy in zygomycosis: a retrospective summary of 91 cases. Clin Infect Dis.

[28] Pagano L, Cornely OA, Busca A, Caira M, Cesaro S, Gasbarrino C, Girmenia C, Heinz WJ, Herbrecht R, Lass-Florl C, Nosari A, Potenza L, Racil Z, Rickerts V, Sheppard DC, Simon A, Ullmann AJ, Valentini CG, Vehreschild JJ, Candoni A, Vehreschild MJ (2013). Combined antifungal approach for the treatment of invasive mucormycosis in patients with hematologic diseases: a report from the SEIFEM and FUNGISCOPE registries. Haematologica.

[29] Reed C, Bryant R, Ibrahim AS, Edwards J, Filler SG, Goldberg R, Spellberg B (2008). Combination polyene-caspofungin treatment of rhino-orbital-cerebral mucormycosis. Clin Infect Dis.

[30] Marty FM, Ostrosky-Zeichner L, Cornely OA, Mullane KM, Perfect JR, Thompson GR, Alangaden GJ, Brown JM, Fredricks DN, Heinz WJ, Herbrecht R, Klimko N, Klyasova G, Maertens JA, Melinkeri SR, Oren I, Pappas PG, Racil Z, Rahav G, Santos R, Schwartz S, Vehreschild JJ, Young JH, Chetchotisakd P, Jaruratanasirikul S, Kanj SS, Engelhardt M, Kaufhold A, Ito M, Lee M, Sasse C, Maher RM, Zeiher B, Vehreschild M, FungiScope Mucormycosis I (2016). Isavuconazole treatment for mucormycosis: a single-arm open-label trial and case-control analysis. Lancet Infect Dis.

[31] Tedder M, Spratt JA, Anstadt MP, Hegde SS, Tedder SD, Lowe JE (1994). Pulmonary mucormycosis: results of medical and surgical therapy. Ann Thorac Surg.

[32] Sun HY, Forrest G, Gupta KL, Aguado JM, Lortholary O, Julia MB, Safdar N, Patel R, Kusne S, Singh N (2010). Rhino-orbital-cerebral zygomycosis in solid organ transplant recipients. Transplantation.

[33] Feng J, Sun X (2018). Characteristics of pulmonary mucormycosis and predictive risk factors for the outcome. Infection.

[34] Brown RB, Johnson JH, Kessinger JM, Sealy WC (1992). Bronchovascular mucormycosis in the diabetic: an urgent surgical problem. Ann Thorac Surg.

[35] Danion F, Aguilar C, Catherinot E, Alanio A, DeWolf S, Lortholary O, Lanternier F (2015). Mucormycosis: new Developments into a Persistently Devastating Infection. Seminars Respir Critical Care Med.

