# Supplementary material for: Mucormycosis in intensive care unit: surgery is a major prognostic factor in patients with hematological malignancy
Source: Ann Intensive Care. 2020 Jun 8;10:74. doi: 10.1186/s13613-020-00673-9 (PMC7280386; doi:10.1186/s13613-020-00673-9)
Supplement: Supplementary file 1 — Additional file 1: Table S1. Demographic characteristics of patients with hematological disease. Table S2. Characteristics of the infection of patients with hematological disease at ICU admission. Table S3. Characteristics of ICU management of patients with hematological disease. [file 13613_2020_673_MOESM1_ESM.docx]

**Additional file 1: Table S1. Demographic characteristics of patients with hematological disease.**

| **Patient** | **Age** | **Sex** | **Body mass index** | **Hematological disease** | **Delay between diagnosis of hematological malignancy and diagnosis of Mucormycosis (days)** | **Allogeneic HSCT (delay before mucormycosis in days)** | **GVH (Grade)** | **Autologous HSCT (delay before mucormycosis in days)** | **Steroids at diagnosis** | **Diabetes** | **Malnutrition** | **Antifungal treatment during the 3 months before mucormycosis** |
| --- | --- | --- | --- | --- | --- | --- | --- | --- | --- | --- | --- | --- |
| **1** | 61 | H | Unknown | Chronic lymphocytic leukemia | 150 | No | NA | No | No | No | Yes | No |
| **2** | 61 | H | 26,2 | B-cell acute lymphoblastic leukemia | 43 | No | NA | No | No | No | No | No |
| **3** | 48 | F | 31,5 | Acute myeloblastic leukemia | 12 | No | NA | No | No | No | No | No |
| **4** | 54 | F | 40 | B-cell acute lymphoblastic leukemia | 638 | No | NA | No | Yes | No | Yes | No |
| **5** | 73 | H | 22 | B-cell acute lymphoblastic leukemia | 20 | No | NA | No | Yes | No | No | Caspofongin |
| **6** | 69 | F | 22 | Acute lymphoblastic leukemia | 21 | No | NA | No | No | No | Yes | Fluconazole |
| **7** | 22 | F | 18,7 | Non-Hodgkin lymphoma | 150 | No | NA | Yes (10) | Yes | Yes | Yes | Isavuconazole |
| **8** | 56 | H | 19 | Myelodysplastic syndrome | 928 | Yes (168) | Yes (Unknown) | No | Yes | No | Yes | Voriconazole |
| **9** | 63 | H | Unknown | Acute myeloblastic leukemia | 348 | Yes (212) | Yes (3) | No | Yes | Yes | No | Voriconazole |
| **10** | 62 | H | 26 | Myelodysplastic syndrome | Unknown | Yes (220) | No | No | Yes | Yes | Yes | No |
| **11** | 34 | H | 20,8 | T-cell Acute lymphoblastic leukemia | 508 | Yes (250) | No | No | No | No | No | Voriconazole and Caspofongin |
| **12** | 54 | H | 23,4 | Non-Hodgkin lymphoma | 157 | No | NA | Yes (3) | Yes | No | No | No |
| **13** | 67 | F | 21,2 | Acute myeloblastic leukemia | 210 | No | NA | No | No | No | No | Amphotericin-B, caspofongin |
| **14** | 33 | H | 20,9 | Hairy cell leukemia | 90 | No | NA | No | No | No | No | No |
| **15** | 16 | F | 22,1 | Acute myeloblastic leukemia | 61 | No | NA | No | No | No | No | No |
| **16** | 45 | H | Unknown | Non-Hodgkin lymphoma B | 21 | No | NA | No | No | No | No | Fluconazole |
| **17** | 16 | F | 22,1 | Acute myeloblastic leukemia | 60 | No | NA | No | No | No | No | No |
| **18** | 29 | F | 17 | Acute myeloblastic leukemia | 806 | Yes (714) | Yes (2) | No | No | No | Yes | No |
| **19** | 38 | H | 22 | Burkitt lymphoma | 90 | No | NA | No | Yes | No | No | Fluconazole |
| **20** | 43 | H | 20,9 | Hodgkin's lymphoma | 707 | Yes (291) | Yes (3) | No | Yes | No | Yes | Posaconazole and Voriconazole |
| **21** | 64 | H | Unknown | Acute myeloblastic leukemia | 255 | Yes (1) | No | No | Yes | No | No | Posaconazole and mycafongin |
| **22** | 44 | H | 23,5 | Acute myeloblastic leukemia | 22 | No | NA | Yes (655) | No | No | No | No |
| **23** | 62 | H | 26 | Acute myeloblastic leukemia | 25 | No | NA | No | No | No | No | Amphotericin-B |
| **24** | 69 | H | 24 | Acute lymphoblastic leukemia | 21 | No | NA | No | Yes | No | No | No |
| **25** | 64 | H | 30 | Acute myeloblastic leukemia | 330 | No | NA | No | No | No | No | Fluconazole, Posaconazole, and caspofongin |
| **26** | 58 | F | 28 | Acute myeloblastic leukemia | 57 | No | NA | No | No | No | No | Caspofongin |
| **27** | 56 | H | Unknown | Hairy cell leukemia | 60 | No | NA | No | No | No | Yes | No |
| **28** | 44 | F | 24 | Acute myeloblastic leukemia | 240 | Yes (120) | Yes (4) | No | Yes | No | No | Voriconazole |
| **29** | 44 | H | Unknown | B-cell acute lymphoblastic leukemia | 26 | No | NA | No | No | No | NC | No |
| **30** | 64 | H | Unknown | Acute myeloblastic leukemia | 60 | No | NA | No | No | No | No | Voriconazole and mycafongin |
| **31** | 37 | H | Unknown | Acute myeloblastic leukemia | 10 | No | NA | No | No | No | No | No |
| **32** | 37 | H | 21,3 | T-cell Acute lymphoblastic leukemia | 677 | Yes (146) | Yes (4) | No | Yes | No | Yes | Posaconazole, Voriconazole and caspofongin |
| **33** | 32 | F | 26 | Acute myeloblastic leukemia | 270 | Yes (134) | Yes (4) | No | Yes | Yes | No | Posaconazole |
| **34** | 58 | H | 25,9 | Non-Hodgkin lymphoma | 169 | No | NA | No | No | No | Yes | Fluconazole |
| **35** | 61 | F | Unknown | T-cell prolymphocytic leukemia | 540 | Yes (80) | Yes (3) | No | Yes | Yes | No | No |
| **36** | 35 | H | Unknown | Non-Hodgkin lymphoma | 3682 | Yes (157) | Yes (4) | Yes (1948) | Yes | Yes | No | Posaconazole |
| **37** | 28 | H | Unknown | Hodgkin's lymphoma | 968 | Yes (unknown) | Yes (4) | Yes (572) | Yes | No | No | Posaconazole |
| **38** | 44 | H | 26,9 | B-cell acute lymphoblastic leukemia | 30 | No | NA | No | No | No | No | Posaconazole |
| **39** | 53 | H | 28,4 | Multiple myeloma | 2144 | Yes (89) | Yes (2) | Yes (1626) | Yes | Yes | Yes | Voriconazole |
| **40** | 47 | H | 20,2 | B-cell acute lymphoblastic leukemia | 305 | Yes (14) | No | No | No | No | Yes | No |
| **41** | 41 | H | 24 | Multiple myeloma | 2188 | Yes (114) | Yes (2) | Yes (1209) | Yes | No | Yes | Fluconazole |

HSCT : hematopoietic stem cell transplantation

**Additional file 1: Table S2. Characteristics of the infection** **of patients with hematological disease at ICU admission**

| **Patient** | **localized,** **contiguous or disseminated mucormycosis** | **Organ(s) involved** | **Isolated fungi** | **IGS2** | **SOFA** | **Delay between symptoms and diagnosis of mucormycosis (days)** | **Delay between symptoms and ICU admission (days)** | **Leukocytes count at ICU admission (G/L)** | **lymphocytes count at ICU admission (G/L)** | **Platelets count at ICU admission (G/L)** |
| --- | --- | --- | --- | --- | --- | --- | --- | --- | --- | --- |
| **1** | localized | lung | Cunninghamella bertholletiae | 55 | 4 | post-mortem | 28 | 110 | NC | 57 |
| **2** | localized | skin | Lichtheimia corymbifera | 35 | 4 | 7 | 1 | 12,2 | 0,2 | 223 |
| **3** | disseminated | lung, sinus , CNS | Lichtheimia corymbifera | 45 | 11 | 20 | 3 | 0,1 | 0 | 19 |
| **4** | contiguous | sinus, skin, CNS, bones | Lichtheimia corymbifera | 52 | 6 | 4 | 6 | 0,4 | <0,4 | 75 |
| **5** | localized | lung | Lichtheimia sp. | 93 | 17 | 1 | 1 | 0,22 | 0 | 52 |
| **6** | localized | lung | Lichtheimia sp. | 58 | 12 | 2 | 2 | 0,19 | 0 | 58 |
| **7** | localized | lung | Lichtheimia sp. | 49 | 7 | 5 | 1 | 0,32 | 0,14 | 15 |
| **8** | disseminated | liver, vessels | Lichtheimia sp. | 40 | 11 | 42 | 41 | 1,5 | 0,2 | 36 |
| **9** | disseminated | lung, skin | Mucor sp. | 55 | 6 | 1 | 1 | 2,7 | 0,1 | 19 |
| **10** | localized | lung | Mucor sp. | 68 | 11 | 5 | 1 | 0,14 | 0 | 13 |
| **11** | localized | lung | Mucor sp. | 41 | 6 | post-mortem | 9 | 0,2 | <0,2 | 30 |
| **12** | disseminated | CNS, skin, spleen, lung, vessels | Rhizomucor sp. | 65 | 10 | post-mortem | 9 | 1,9 | 0,1 | 49 |
| **13** | localized | lung | Unknown | 71 | 15 | 7 | 3 | 0 | 0 | 19 |
| **14** | localized | sinus | Unknown | 34 | 5 | 6 | 6 | 0,2 | 0,1 | 43 |
| **15** | localized | lung | Unknown | 18 | 1 | 29 | 29 | 9,9 | 0,4 | 213 |
| **16** | localized | abdomen | Unknown | 53 | 12 | 17 | 1 | 0,14 | 0 | 36 |
| **17** | localized | lung | Unknown | 18 | 0 | 29 | 29 | 9,9 | 0,43 | 213 |
| **18** | localized | lung | Rhizomucor miehei | 42 | 7 | 28 | 25 | 3,1 | 0,2 | 270 |
| **19** | contiguous | liver, diaphragm, stomach | Rhizomucor pusillus | 73 | 8 | 3 | 1 | 0,1 | 0 | 76 |
| **20** | localized | lung | Rhizomucor pusillus | 80 | 12 | 11 | 1 | 0,5 | 0 | 5 |
| **21** | disseminated | lung, skin | Rhizomucor pusillus | 94 | 15 | 4 | 2 | 0,02 | 0 | 58 |
| **22** | localized | lung | Rhizomucor sp. | 41 | 7 | 6 | 19 | 7,7 | NC | 44 |
| **23** | localized | lung | Rhizomucor sp. | 67 | 11 | 3 | 1 | 2,3 | NC | 11 |
| **24** | disseminated | skin, CNS, kidney, spleen | Rhizomucor sp. | 64 | 8 | 2 | 1 | 0,2 | 0 | 27 |
| **25** | disseminated | CNS, kidney, skin | Rhizomucor sp. | 88 | 16 | 1 | 1 | 0,12 | 0 | 16 |
| **26** | localized | lung | Rhizomucor sp. | 98 | 16 | post-mortem | during ICU stay | 0 | 0 | 14 |
| **27** | disseminated | lung, CNS | Rhizomucor sp. | 59 | 6 | 9 | 4 | 0,58 | 0,4 | 4 |
| **28** | localized | skin | Rhizopus sp. | 25 | 2 | 1 | 13 | 5,52 | NC | 59 |
| **29** | contiguous | lung, pleura, pericard | Rhizopus sp. | 32 | NC | 23 | 24 | NC | NC | NC |
| **30** | localized | lung | Rhizopus sp. | 66 | 12 | 21 | 7 | 0,2 | 0,1 | 56 |
| **31** | localized | lung | Rhizopus sp. | 35 | 9 | 7 | 1 | 3,5 | NC | 85 |
| **32** | localized | skin | Rhizopus oryzae | 26 | 6 | 5 | during ICU stay | 7,89 | 0,84 | 101 |
| **33** | contiguous | lung, sinus | Rhizopus oryzae | 49 | 8 | 8 | 1 | 0,5 | 0,04 | 63 |
| **34** | contiguous | sinus, skin, nose and mouth | Rhizopus oryzae | 58 | 13 | 16 | 9 | 0,1 | <0,1 | 21 |
| **35** | disseminated | lung, liver | Rhizopus oryzae | 59 | 10 | 9 | 10 | 12,24 | 0,7 | 18 |
| **36** | disseminated | lung, mediastinum, abdomen | Rhizopus sp. | 74 | 10 | 5 | 4 | 1,39 | 0,03 | 13 |
| **37** | localized | lung | Rhizopus sp. | 23 | 5 | 28 | 28 | 14,4 | 0,68 | 112 |
| **38** | localized | lung | Rhizopus sp. | 53 | 12 | 5 | 1 | 0,1 | 0 | 9 |
| **39** | contiguous | lung, skin, mouth | Rhizopus sp. | 52 | 13 | 9 | 9 | 1,7 | 0,05 | 14 |
| **40** | localized | lung | Rhizopus sp. | 78 | 10 | 10 | during ICU stay | 3 | 0 | 12 |
| **41** | localized | lung | Rhizopus sp. | 83 | 12 | 6 | during ICU stay | 1,6 | 0,03 | 25 |

CNS : central nervous system

**Additional file 1:Table S3. Characteristics of ICU management of patients with hematological disease .**

| **Patient** | **Antifungal therapy** | **Delay between symptoms and efficient treatment (days)** | **Delay between symptoms and curative surgical management (days)** | **Mechanical ventilation** | **Renal replacement therapy** | **Granulocyte-colony stimulating factor** | **Length of ICU stay (days)** | **Survival in ICU** |
| --- | --- | --- | --- | --- | --- | --- | --- | --- |
| **1** | Posaconazole | 10 | No surgery | Yes | No | Yes | 24 | No |
| **2** | L-AmB + Posaconazole | 8 | No surgery | No | No | No | 3 | Yes |
| **3** | L-AmB | 20 | No surgery | Yes | No | Yes | 50 | No |
| **4** | L-AmB | 5 | 6 | Yes | No | No | 5 | No |
| **5** | No treatment | No treatment | No surgery | Yes | No | No | 2 | No |
| **6** | No treatment | No treatment | No surgery | Yes | No | No | 2 | No |
| **7** | L-AmB + Caspofungin | 0 | No surgery | Yes | Yes | No | 16 | No |
| **8** | L-AmB | 0 | No surgery | Yes | No | No | 11 | No |
| **9** | L-AmB + Posaconazole | 9 | No surgery | Yes | No | No | 17 | No |
| **10** | No treatment | No treatment | No surgery | Yes | No | No | 5 | No |
| **11** | No treatment | No treatment | No surgery | Yes | Yes | No | 6 | No |
| **12** | L-AmB | 11 | No surgery | Yes | No | No | 8 | No |
| **13** | L-AmB | 7 | No surgery | Yes | No | No | 21 | No |
| **14** | L-AmB | 4 | 5 | No | No | Yes | 3 | Yes |
| **15** | L-AmB + Caspofungin | 4 | 29 | No | No | No | 6 | Yes |
| **16** | L-AmB | 22 | 17 | Yes | Yes | No | 34 | No |
| **17** | L-AmB + Caspofungin | 4 | 29 | No | No | No | 6 | Yes |
| **18** | L-AmB | 11 | 25 | Yes | No | No | 8 | Yes |
| **19** | L-AmB + Caspofungin | 4 | 13 | Yes | Yes | No | 23 | No |
| **20** | L-AmB | 2 | No surgery | Yes | Yes | Yes | 26 | No |
| **21** | Posaconazole | 1 | No surgery | Yes | Yes | No | 3 | No |
| **22** | L-AmB | 6 | 19 | No | No | No | 2 | Yes |
| **23** | L-AmB + Posaconazole | 0 | No surgery | Yes | No | No | 19 | No |
| **24** | L-AmB + Caspofungin | 2 | No surgery | Yes | No | No | 23 | No |
| **25** | L-AmB | 1 | No surgery | Yes | No | No | 3 | No |
| **26** | No treatment | No treatment | No surgery | Yes | Yes | Yes | 10 | No |
| **27** | L-AmB | 9 | No surgery | Yes | No | No | 9 | No |
| **28** | L-AmB | 0 | 1 | Yes | No | No | 35 | No |
| **29** | L-AmB | 23 | 111 | No | No | No | 8 | Yes |
| **30** | L-AmB | 29 | No surgery | Yes | Yes | Yes | 24 | No |
| **31** | No treatment | No treatment | No surgery | Yes | No | No | 7 | No |
| **32** | L-AmB | 5 | 6 | Yes | Yes | No | 24 | No |
| **33** | L-AmB | 9 | No surgery | Yes | No | Yes | 11 | No |
| **34** | L-AmB | 16 | 21 | Yes | Yes | No | 12 | No |
| **35** | L-AmB + Caspofungin | 11 | No surgery | Yes | Yes | No | 6 | No |
| **36** | Caspofungin | 5 | No surgery | Yes | No | No | 9 | No |
| **37** | L-AmB + Caspofungin | 8 | No surgery | Yes | No | No | 7 | No |
| **38** | No treatment | No treatment | No surgery | Yes | No | No | 2 | No |
| **39** | L-AmB | 13 | No surgery | Yes | Yes | No | 12 | No |
| **40** | No treatment | No treatment | No surgery | Yes | No | No | 5 | No |
| **41** | No treatment | No treatment | No surgery | Yes | No | No | 2 | No |

L-AmB : liposomal Amphotericin-B
